# Extracellular vesicle-mediated transfer of miR-21-5p from mesenchymal stromal cells to neurons alleviates early brain injury to improve cognitive function via the PTEN/Akt pathway after subarachnoid hemorrhage

**DOI:** 10.1038/s41419-020-2530-0

**Published:** 2020-05-13

**Authors:** Xiao Gao, Ye Xiong, Qizhao Li, Min Han, Dezhi Shan, Guozheng Yang, Shouji Zhang, Danqing Xin, Rongrong Zhao, Zhen Wang, Hao Xue, Gang Li

**Affiliations:** 1 Department of Neurosurgery, Qilu Hospital, Cheeloo College of Medicine, Shandong University, 107 Wenhua Xi Road, Jinan, Shandong Province China; 2 Institute of Brain and Brain-Inspired Science, Shandong University, 44 Wenhua Xi Road, Jinan, Shandong Province 250012 P.R. China; 3 Shandong Provincial Key Laboratory of Brain Function Remodeling, Jinan, Shandong Province China; 4 Department of Neurosurgery, The First Affiliated Hospital of Wenzhou Medical University, Wenzhou, Zhejiang Province China; 5 Department of Physiology, Shandong University School of Basic Medical Sciences, Jinan, China

**Keywords:** Stroke, Experimental models of disease

## Abstract

Patients with subarachnoid hemorrhage (SAH) often suffer from cognitive function impairments even when they have received proper treatment, such as the clipping or coiling of aneurysms, and this causes problems with returning to work and burdens the family. Increasing attention has been paid to mesenchymal stem cell (MSC)-derived extracellular vesicle (MSC-EV) as promising therapeutic vesicles for stroke management. In this study, we explored the potential role of MSC-EV in a rat model of SAH. We observed that MSC-EV ameliorated early brain injury (EBI) after SAH by reducing the apoptosis of neurons and that SAH induced an increase in the expression level of miR-21 in the prefrontal cortex and hippocampus. In addition, using miRNA profiling and CSF sequencing data from the exRNA Atlas, we demonstrated that EV-derived miR-21 protected neurons from apoptosis and alleviated SAH-induced cognitive dysfunction. The neuroprotective role of MSC-EV was abrogated by miR-21 knockdown or the administration of MK2206, a PTEN/Akt inhibitor. Overall, our results suggest that MSC-EV promotes neuronal survival and alleviates EBI after SAH through transferring miR-21 to recipient neurons.

## Introduction

Subarachnoid hemorrhage (SAH) is a life-threatening neurological disease with high mortality and disability rates^1,2^. However, those patients who survive acute bleeding and rebleeding often suffer from cognitive deficits with decreased life quality for years after SAH^3–5^. Increasing evidence indicates that early brain injury (EBI) may account for the poor outcomes observed in SAH patients^6–8^.

Recently, an increasing amount of attention has been paid to mesenchymal stem cell (MSC)-derived EVs, as messengers that function in intercellular communication and promising therapeutic vesicles used for regeneration medicine, which are believed to be able to replace MSC cell therapy^9^. In addition, theoretically, using secreted EVs rather than cells themselves could overcome some safety problems with MSC therapy. EVs contain proteins, lipids, mRNAs, and noncoding RNAs that can modulate the pathophysiological state of receiver cells. MSC EV-based therapy has shown promise in experimental models of liver injury^10,11^, renal injury^12^, acute myocardial infarction^13^, wound healing^14^, and so on.

MSC cell therapy has been used in the restorative process after stroke and traumatic brain injury (TBI) since the early 2000s. Additionally, much evidence has suggested that neuroprotective effects are mediated by EVs secreted by MSCs. For example, Michael Chopp et al. demonstrated that MSC EVs could be taken up by neural cells. These miR-133b-enriched EVs could help restore neurological function^15,16^. However, only a few studies concerning the role of MSC therapy in SAH have been reported, and these have demonstrated that the neuroprotective effects partly resulted from the alleviation of microglia-mediated neuroinflammation^17,18^. However, whether MSC EVs play a part in SAH therapy remains unclear.

MiRNA is one of the important cargoes encapsulated in EVs. These miRNAs serve as messengers for intercellular communications, biomarkers for clinical diagnosis and prognosis, and therapeutic agents for diseases. Bache et al. reported that miRNA profile changed after SAH^19^, while they didn’t explore the mechanisms of miRNA upregulation.

The aim of this study was to investigate the therapeutic potential of MSC EVs in SAH, especially for cognitive function recovery, and to explore the underlying mechanisms. We demonstrated that MSC EVs can alleviate EBI and improve cognitive function after SAH and that the mechanism involved mainly depends on the transfer of MSC-derived EV-derived miR-21-5p to neurons to activate AKT signaling to inhibit apoptosis.

## Materials and methods

### MSC-EV isolation and characterization

Bone marrow-derived MSCs were obtained from Cyagen Bioscience (Suzhou, China) and cultured with a-modified MEM medium containing 10% fetal bovine serum (FBS) and penicillin–streptomycin in 175 cm^2^ tissue culture flasks. For EVs isolation, we replaced the conventional culture medium with 20 ml medium containing 10% EV-depleted FBS (Vivacell Biotechnology, Germany) when the cells reached 60–80% confluence. Following an additional 48 h of culturing, the media from about 5–8 × 10^6^ cells were then collected for centrifugation^20^. The EVs were stored at −80 °C.

The concentration of EVs was determined by using Enhanced BCA Protein Assay Kit (Beyotime). 40 ml culture medium from two T175 flasks contained about 600 μg of EV. To determine the size distribution of the EVs, nanoparticle tracking analysis was performed using the qNano system (Izon) on samples diluted with PBS. And antibodies of EV markers and negative controls were used for western blot analysis: TSG101 (1:1000, Cell Signaling), CD9 (1:1000, Cell Signaling) and Calnexin (1:1000, Cell Signaling).

### SAH rat model

The intravascular perforation SAH model was induced as previously described^21,22^. Briefly, adult male SD rats weighing 300–320 g were anesthetized through an intraperitoneal injection of chloral hydrate (340 mg/kg body weight). After the left carotid artery and associated branches were dissected, a 3–0 monofilament nylon suture was inserted into the stretched external carotid artery, then guided into the internal carotid artery wall and was withdrawn following the perforation. Rats in the sham group underwent the same surgical procedure without perforation. This study was performed in accordance with relevant guidelines and approved by the Ethics Committee of Qilu Hospital.

### Experimental design

All animal experiments underwent randomization at entry. Animal subjects were randomly assigned to treatment groups and during analysis. For immunostaining imaging experiments, the experimenter was blinded to the slides selected for imaging on microscopy.

#### Experimental design 1

To determine the effects of MSC-EV on EBI after SAH, 48 rats (59 rats were used, but only 48 rats survived after the surgery) were randomly assigned into three groups: sham, SAH + PBS and SAH + MSC-EV (*n* = 16). All rats were sacrificed at 48 h after SAH for assays including neurological score assessment, brain water content, western blot, and histopathological observation.

#### Experimental design 2

Nine rats were used to evaluate the distribution of PKH67-labeled MSC-EV administered by i.v. throughout the whole body. Nine rats (15 rats were used, but only 9 rats survived) were randomly assigned to three groups: sham, SAH + PBS and SAH + MSC-EV (*n* = 3). Twenty-four hours after MSC-EV administration, all rats were deeply anesthetized and perfused with 4% paraformaldehyde prior to frozen sectioning.

#### Experimental design 3

A total of 32 rats (41 rats were used, but only 32 rats survived) were used in this cohort, in which rats were randomly divided into eight subgroups: sham, 3, 6, 12, 24, 48, 72 h and 1-week post-SAH (*n* = 4). Each group of rats was sacrificed at the indicated time post SAH for RNA extraction from the prefrontal cortex and hippocampus.

#### Experimental design 4

To explore the molecular mechanism underlying neuroprotection by MSC-EV, we employed miR-21-5p inhibitors and MK2206 to block its therapeutic effects. Fifty rats (63 rats were used, but only 50 rats survived) were randomly divided into the following groups: sham, SAH + PBS, SAH + MSC-EV, SAH + MSC-EV(-21) and SAH + MSC-EV + MK2206. Each group of rats was sacrificed at 48 h after SAH for neurological function, western blot, HE staining, TUNEL assay and double immunostaining of NeuN and cleaved caspase-3.

#### Experimental design 5

Thirty rats (36 rats were used, but only 30 rats survived) were used to assess cognitive function impairments after SAH. The Morris Water Maze task was performed to evaluate cognitive changes as previously described^23^ in all groups of rats, including the sham, SAH + PBS, and SAH + MSC-EV groups (*n* = 10).

### EVs tracing

EVs were dyed with PKH67 according to the manufacturer’s protocol. In brief, 500 μg MSC-EVs were incubated with 4 µl PKH67 diluted in 1 ml dyeing buffer for 15 min, then 2 ml of stopping buffer containing 0.5% bovine serum albumin (BSA) was added, and the labeled EVs were washed by centrifugation at 100,000 × *g* for 4 h. The labeled EVs were resuspended with PBS before administration. As negative control, 4 μl PKH26/ PKH67 dye was added to 1 ml dyeing buffer and incubated with equivalent volume of PBS for 15 min. And this collection containing little free dye from ultracentrifugation was injected into SAH rats as control.

### Neurological scoring

At 48 h after SAH, we assessed neurobehavioral function according to the modified Garcia scoring system^24,25^. In brief, this system included six tests: spontaneous activity (0–3 points), reaction to side stroking (1–3 points) and to vibrissae touch (1–3 points), limb symmetry (0–3 points), forelimb outstretching (0–3 points), and climbing (0–3 points). The total scores on these tests ranged from 3 to 18^26,27^. Higher scores indicated better neurological behavior.

### Brain water content

The brains were quickly dissected from the skulls 48 h after SAH and then weighed before and after 48 h of heating at 95 °C. Brain edema was calculated as (wet weight – dry weight)/wet weight × 100%.

### TUNEL assay

Apoptosis was detected using an in-situ cell death detection kit (Roche) according to the manufacturer’s protocol. After counterstaining with DAPI, the slides were kept in Antifade Mounting Medium (Beyotime). Three random microscope fields (×20) were imaged for every slide of brain tissue.

### RNA extraction and qRT-PCR

Total RNA was extracted from the prefrontal cortex and hippocampus using TRIzol (Invitrogen) according to the manufacturer’s instructions. 1 µg RNA was reverse-transcribed into cDNA using the ReverTra Ace qRT-PCR kit (Toyobo). Real-time PCR was performed using the SYBR Premix MASTER Kit (Roche) with a LightCycler 480 Instrument (Roche). All data for each sample were collected in triplicate. Standard curves were generated, and the relative amount of miRNA was normalized to the amount of U6 (2^−ΔΔCt^).

### Western blot analysis

Western blot analysis was performed as described previously^23^. The protein concentration was determined using an Enhanced BCA Protein Assay Kit (Beyotime). Protein samples (30–50 µg) were loaded onto 10% or 12% SDS-polyacrylamide gel for electrophoresis. Then polyvinylidene difluoride membranes (Millipore) were used for protein transfer and incubated with the following primary antibodies: Bax (1:2000, Abcam), Bcl-2 (1:1000, Abcam), cleaved caspase-3 (1:500, Cell Signaling), phospho-Akt (Ser473) (1:500, Cell Signaling), Akt (1:1000; Cell Signaling), and phosphatase and tensin homolog deleted on chromosome 10 (PTEN) (1:500, Cell Signaling). GAPDH (1:2000, Sigma-Aldrich) was used as an internal control. Horseradish peroxidase conjugated to either goat anti-mouse or rabbit IgG was used as a secondary antibody (1:5000, Cell Signaling). The membranes were detected using ChemiDoc XRS+ (Bio-Rad).

### Immunofluorescence imaging

The slides were fixed in 4% paraformaldehyde for 15 min and blocked with 10% goat serum in PBS. The slides were incubated overnight in a humidified chamber at 4 °C with the following primary antibodies: NeuN, 1:500, Abcam; cleaved caspase-3, 1:100, Cell Signaling. After primary antibody incubation, the samples were washed with PBS and incubated with the matching fluorescent-conjugated secondary antibody (1:500 dilution, Thermo Fisher) at room temperature for 1 h. Images were captured using a Leica DMi8 microscope. Three microscope fields (×20) showing active caspase-3/NeuN double-positive cells were chosen and imaged. The number of active caspase-3/NeuN double-positive cells was calculated as the mean of the numbers of cells counted in six images from each rat. Four rats were included for the staining of each group. Counting was performed in a blinded manner.

### Fluorescence in situ hybridization (FISH)

Rno-miR-21-5p probe (sequences: 5′-TCAACATCAGTCTGATAAGCTA-3′) was synthesized by GenePharma (Shanghai, China) and used under the instructions of miRNA FISH kit (GenePharma). Briefly, On day 1, the frozen sections were rehydrated and digested by proteinase K for 20 min at 37 °C. Next, sections underwent pretreatment with denaturing solution for 8 min at 78 °C. The sections were then incubated with miR-21-5p-5p probe overnight at 37 °C. On day 2, the sections were rinsed in pre-warmed washing solution at 43 °C and PBS after hybridization. Thereafter, sections were blocked using 5% bovine serum albumin (BSA) for 60 min at 37 °C and incubated overnight at 4 °C with NeuN antibody. On day 3, sections were incubated with the fluorescent secondary antibody for 1 h at room temperature in a dark room. The nuclei were counterstained with DAPI.

### Intracerebroventricular drug administration

Briefly, rats were placed in a stereotaxic apparatus under inhalation anesthesia. 100 μg MK2206 in 5 μl DMSO was slowly injected into left ventricle with a 10-μl Hamilton syringe (Hamilton, USA) through a drilling hole according to the following coordinates relative to bregma: 1.5 mm posterior and 1.0 mm lateral and 3.5 mm deep.

### Statistical analysis

SPSS 20 and GraphPad Prism were used for the statistical analysis and graph plotting, respectively. The neurological scores by modified Garcia scoring system were analyzed by Kruskal–Wallis one-way analysis of variance (ANOVA) and Dunn’s post hoc test. In addition, the test data are presented as the mean ± SD and analyzed by one-way ANOVA followed by Tukey’s post hoc analysis for multiple comparisons of means. When *p* < 0.05, the difference was considered statistically significant.

## Results

### MSC-EV attenuated neurological deficits after SAH

**Fig. 1:**
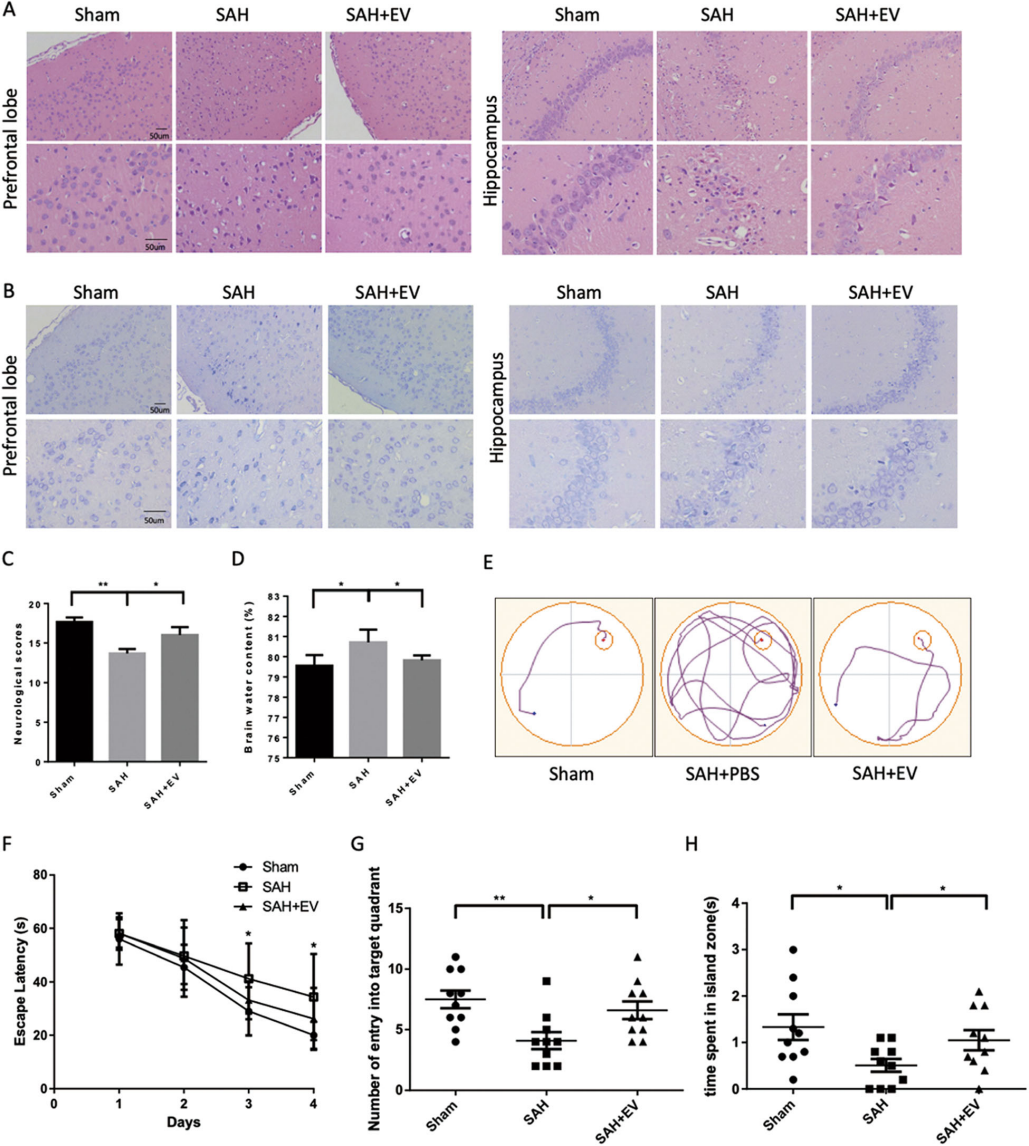
MSC-EV attenuated neurological deficits after SAH. **a**, **b** Hematoxylin and eosin (HE) and Nissl staining of the prefrontal cortex and hippocampus at 48 h after SAH (*n* = 6). **c** Neurological behavior scores in each group (*n* = 12). **d** Brain water content was measured 48 h after SAH (*n* = 6). **e** Representative tracing images of the swimming path of each group. **f** The escape latency in acquisition trials from the Morris Water Maze test. **g** Number of entries into the target quadrant and **h** time spent in the island zone in the probe trail were recorded and analyzed (*n* = 10).

MSC-EV was isolated from FBS-depleted culturing medium of MSC and characterized by qNano and western blotting of EV protein markers (Fig. S1). To determine the effects of MSC-EV, 100 µg of EVs were slowly injected into the caudal vein of each SAH rat 1 h post bleeding, while the control group rats were administered an equivalent volume of phosphate-buffered saline (PBS). Compared with the SAH + PBS group, the SAH + EV group achieved higher neurological scores at 48 h after SAH insult, implying improved neurological performance (Fig. 1c). The brain water content also decreased after MSC-EV administration, as shown in Fig. 1d. Brain injury was also determined by HE staining (Fig. 1a) and Nissl staining (Fig. 1b). Many more neurons exhibited a contracted appearance in the SAH group, while MSC-EV alleviated this type of damage.

Moreover, the Morris Water Maze (MWM) test was performed to evaluate cognitive functional impairment in a relatively long term after SAH. Two weeks after perforation, a four-day hidden platform test and a one-day probe test were performed successively. Compared with the sham group, the SAH group showed obviously impaired cognitive function and learned more slowly, while MSC-EV improved cognitive behavior and shortened the time spent seeking the hidden island (Fig. 1f). Furthermore, in the probe test, SAH rats treated with MSC-EV entered the target quadrant more often and stayed in the island zone longer than SAH rats treated with PBS (Fig. 1g and h), indicating that a stronger memory was obtained.

### MSC-EV inhibited neuronal apoptosis resulting from EBI

**Fig. 2:**
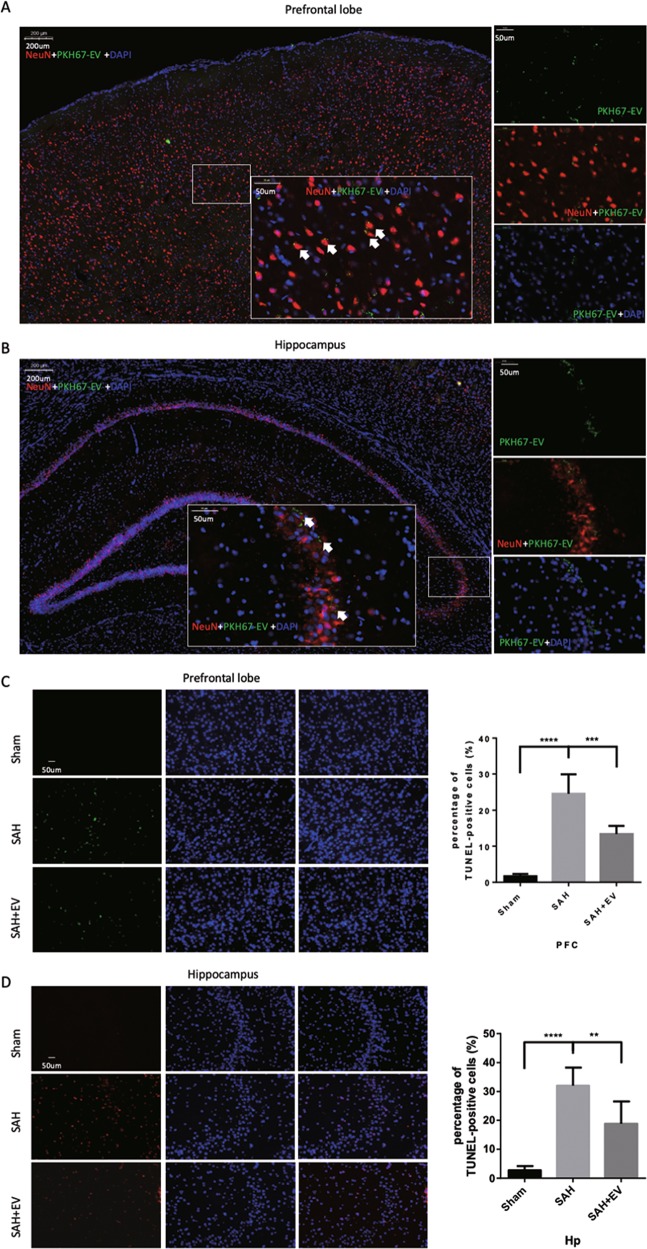
MSC-EV target injured neurons and inhibit apoptosis after SAH. **a**, **b** Representative fluorescence images of brain sections stained with neuronal (NeuN) marker 48 h after SAH. PKH67-dyed EVs were administered by i.v. into SAH-affected rats (*n* = 3). **c**, **d** TUNEL staining of the cerebral cortex and hippocampus, respectively.

**Fig. 3:**
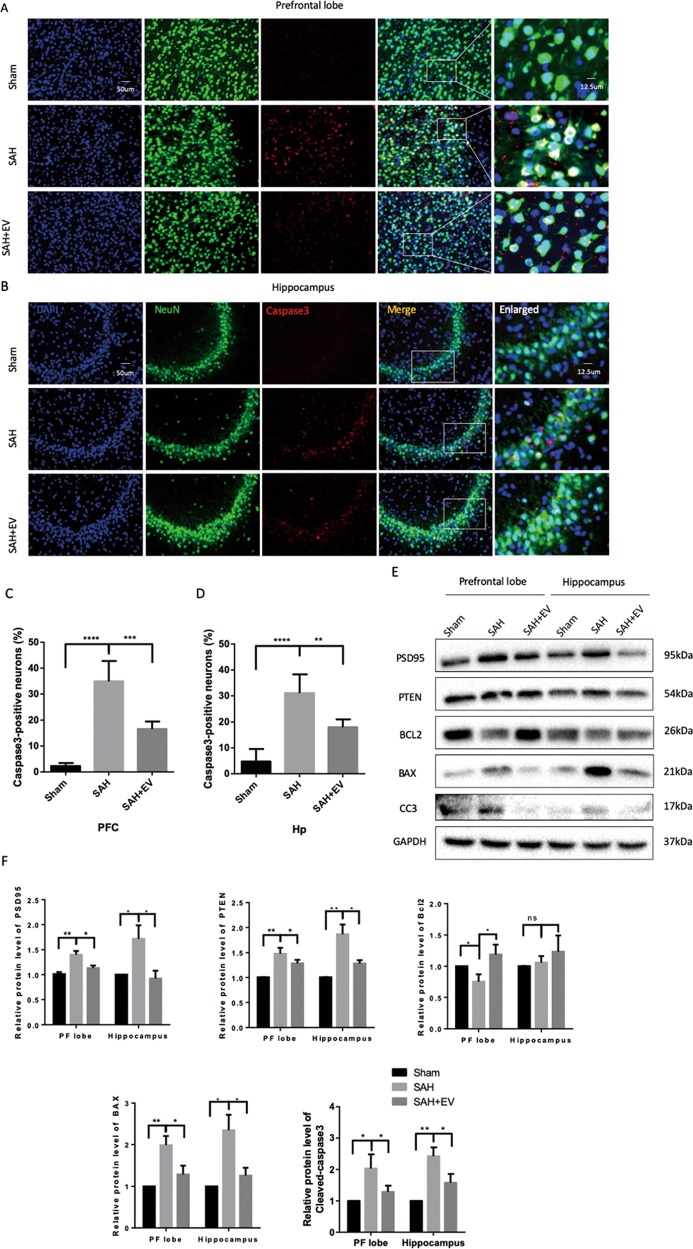
MSC-EV inhibited neuronal apoptosis to alleviate EBI. **a**, **b** Double immunofluorescence staining of cleaved-caspase-3 and NeuN in the prefrontal cortex and hippocampus, respectively. Quantitative analysis of immunofluorescence staining of cleaved-caspase-3 for the prefrontal cortex and hippocampus (**c, d**). **e** Western blot analysis of important apoptosis-related proteins. **f** Quantitative analysis of western blot with GAPDH.

To investigate the protective mechanism of MSC-EV, we performed EV tracing assays using PKH67-dyed EVs. After sacrificing the animals, brain sections were obtained and further stained with neuronal or astrocytic markers to determine which types of cells took up the EVs. As shown in Figs. 2a, b and S3, most EVs were taken up by neurons, while little free dye couldn’t target damaged brain region in control group (Fig. S2). In addition, PKH67-dyed EVs were also found in the liver, kidney, and spleen of SAH rats (Fig. S4). Meanwhile, the number of TUNEL-positive neurons was increased 48 h after SAH, and the number of apoptotic cells in the cortex and hippocampus were reduced significantly after treatment with MSC-EV (Figs. 2c, d and S5). Caspase 3/NeuN staining was also used to verify SAH-induced neuronal apoptosis. As shown in Fig. 3a and b, MSC-EV administration significantly inhibited neuronal apoptosis after SAH. In addition, the protective role of MSC-EV was demonstrated by western blot (Fig. 3e). The expression of Bcl-2 increased as a result of the effects of MSC-EV, while the expression of Bax and cleaved-caspase-3 decreased compared with that in the SAH-PBS group. Consistent with the results of our previous studies, PSD95 and PTEN were upregulated after SAH onset^23,28^, and MSC-EV alleviated the pathological accumulation of both targets. PSD95 participates in the formation of synapses, while its role in SAH still remains unclear.

### miRNA sequencing showed potential key targets in MSC-derived EVs

**Fig. 4:**
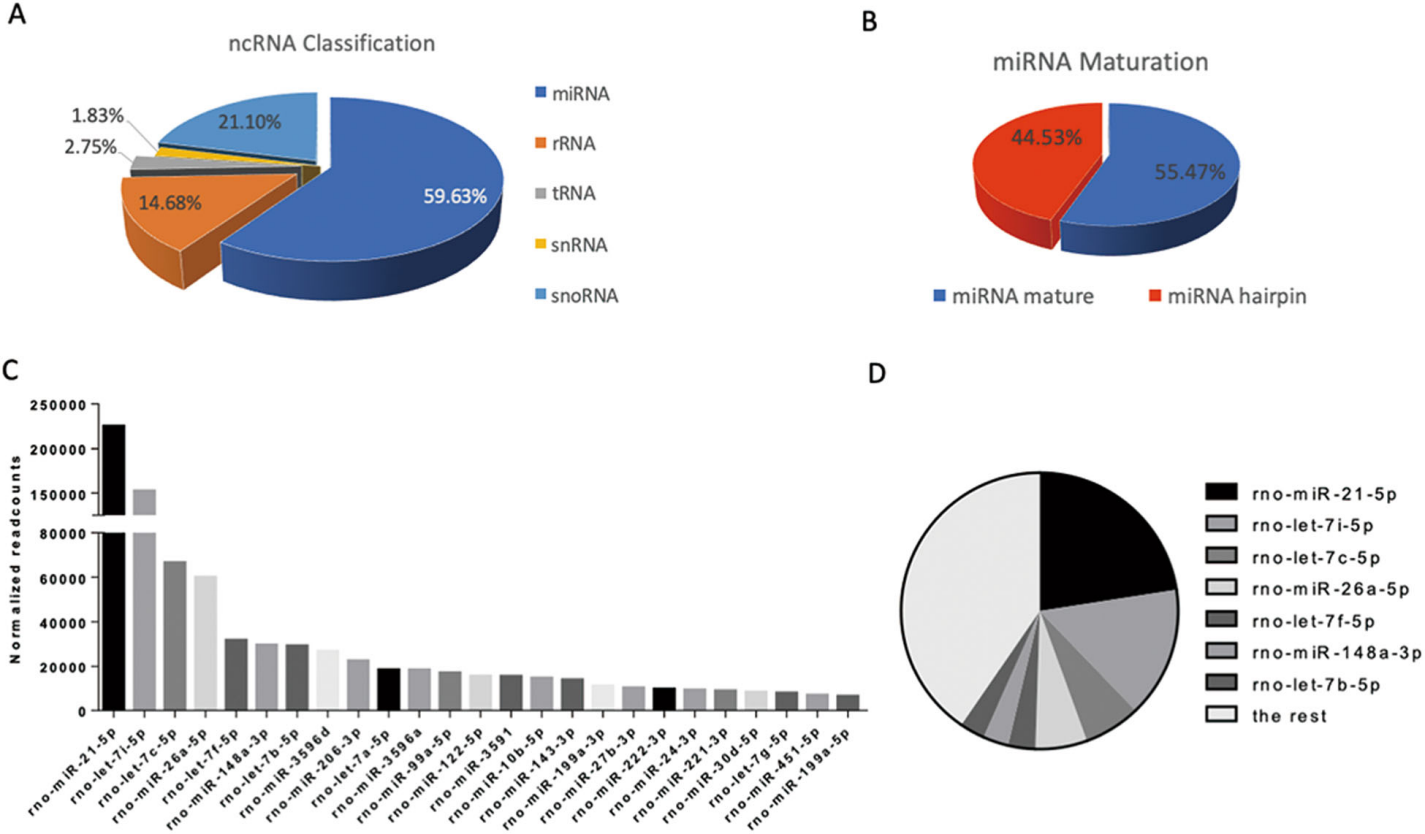
miRNA sequencing revealed potential key targets in MSC-derived EVs. **a** Non-coding RNA sequencing revealed microRNA is the largest portion. **b** miRNA maturation analysis indicated that about half miRNA was single-chain mature form. **c** Total numbers of reads representing the top 25 miRNAs according to miRNA profiling of MSC-derived EVs are shown. **d** The top 7 miRNAs accounted for 59.6% of the total miRNA in MSC EVs.

**Fig. 5:**
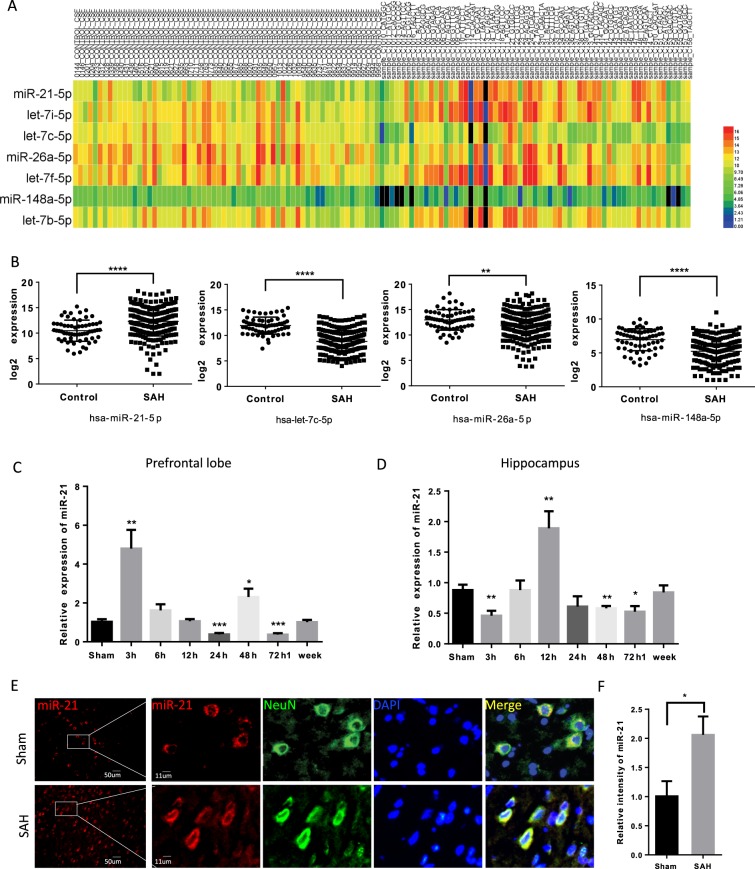
SAH induced miR-21 upregulation, which wore down along time. **a** Heat map showing miRNA expression changes in CSF after SAH determined via comparison with healthy donors. **b** Dot plot showing the differential expression levels of the top 4 miRNAs. **c**, **d** Time course of mir-21 expression in rat brain tissues after SAH (*n* = 4). **e** Representative images of the immunostaining of in situ mir-21 expression in NeuN-positive cortical neurons. **f** Quantitative analysis of in situ miR-21 expression.

MicroRNA is one of the most prominent types of content in EVs and plays an important role in EV-mediated intercellular communication. Because of this, we used high-throughput sequencing to identify miRNA species and their abundance in SD-MSC-derived EVs. According to the sequencing data, miRNA makes up above half of total non-coding RNA in EVs (Fig. 4a), 55.47% of which is in single-chain mature form (Fig. 4b). As shown in Fig. 4c and d, miR-21-5p, along with the other top 6 miRNAs in terms of abundance, constitute nearly 60% of the miRNA contents in EVs. And miR-21-5p alone accounts for as much as 22.5% of contents. In the meantime, the let7 miRNA family drew much attention from us, with the four of let-7i, let-7c, let-7f, and let-7b lied in top 7 miRNAs. However, according to sequencing data (5UH2TR000891-02) derived from SAH patient cerebrospinal fluid (CSF) from the *exRNA* Atlas^29^, miR-21-5p expression increased significantly after SAH compared with that found in healthy donors (Fig. 5a, b, and S6), while the expression of let-7 family didn’t exhibit consistency. We also performed qRT-PCR to determine the expression of other miRNAs in prefrontal cortex and hippocampus 48 h post SAH (Fig. S7). Considering the above, we supposed that miR-21-5p may mediate the neuroprotective role of MSC-EV.

### SAH induced miR-21-5p upregulation

Dozens of miRNAs have been shown to be critical for the development of neurological diseases^30^, among which miR-21-5p has been extensively studied^31–34^.

PCR was performed to assess the time course of miR-21-5p expression after SAH. As shown in Fig. 5c, the expression level of miR-21-5p increased and peaked at 3 h post SAH. However, it was decreased at 3 h and increased at 12 h in the hippocampus (Fig. 5d). The expression and distribution of miR-21-5p was further identified by FISH 48 h after SAH. As shown in Fig. 5e, compared with the sham group, miR-21-5p expression was increased in the cortex 48 h after SAH in the SAH group. In addition, the increases in miR-21-5p were mostly observed in NeuN-positive cells (Fig. 5e). Thus, we assume that miR-21-5p expression was upregulated and played a protective role in neurons after SAH but decreased somehow over time, while MSC-EV administration could reverse the loss of miR-21-5p to inhibit damage.

### MiR-21-5p mediated the neuroprotective effects of MSC-EV

**Fig. 6:**
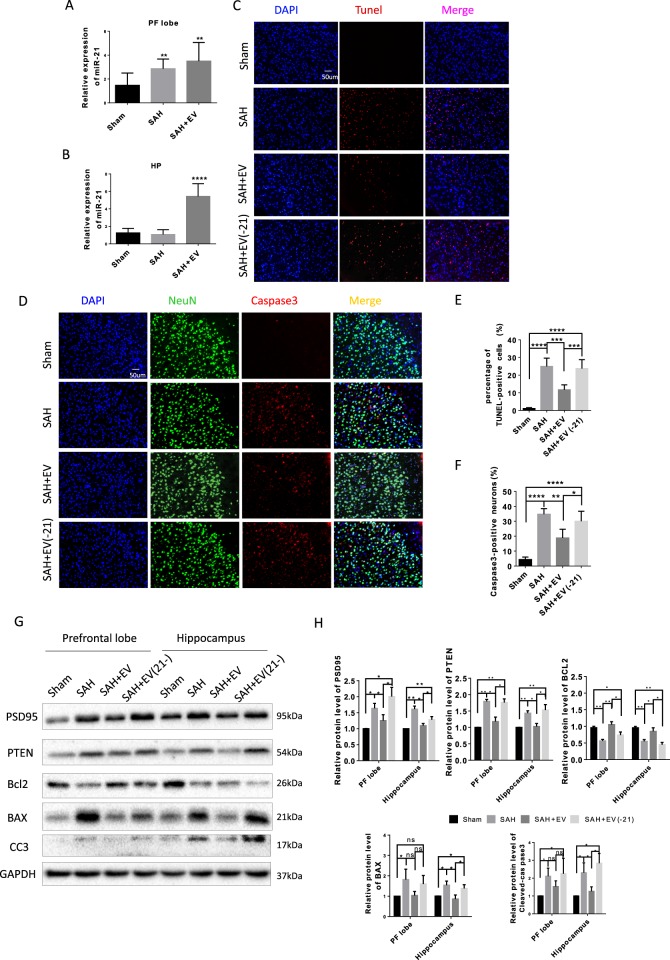
miR-21 mediated the neuroprotective effects of MSC-EV. **a**, **b**, MSC-EV increased mir-21 expression in brain tissues 48 h after SAH. MSC-EV were administered to SAH rats in the presence of miR-21 knockdown. TUNEL staining (**c**) and double immunofluorescence staining (**d**) of cleaved-caspase-3 and NeuN in the prefrontal cortex 48 h after SAH (*n* = 4). Quantitative analyses were performed for TUNEL staining (**e**) and immunofluorescence staining (**f**). **g** Western blot detected the protein level of PSD95, PTEN, BCL2, BAX, and cleaved-caspase3 in prefrontal lobe and hippocampus. (*n* = 3). **h** Protein levels were normalized to that of GAPDH.

First, we detected the expression of assumed-protective miR-21-5p. In addition, miR-21-5p expression was examined by qRT-PCR at 48 h after SAH. As shown in Fig. 6a and b, miR-21-5p expression in the prefrontal cortex increased significantly, while its expression in the hippocampus decreased without significance at 48 h after SAH. As expected, during treatment with MSC-EV, miR-21-5p expression increased significantly, implying that the neuroprotective effects of MSC-EVo were mediated by miR-21-5p.

To verify the importance of miR-21-5p, we transfected synthesized miR-21-5p inhibitors in MSCs to block miR-21-5p activity in EVs. The blocking effect was detected by a TUNEL assay (Fig. 6c) and caspase3/NeuN staining (Fig. 6d). The MSC-EV(-21) failed to inhibit neuronal apoptosis caused by SAH. In addition, western blot results also showed that miR-21-5p inhibitors effectively suppressed the anti-apoptotic effects of MSC-EV on neurons after SAH (Fig. 6g). The PTEN/PI3K-AKT signaling pathway has been widely studied as a downstream of miR-21-5p in many pathophysiological processes. In the context of SAH, we discovered that PTEN expression was upregulated and suppressed by EV-derived miR-21-5p from cultured MSCs (Fig. 6g).

**Fig. 7:**
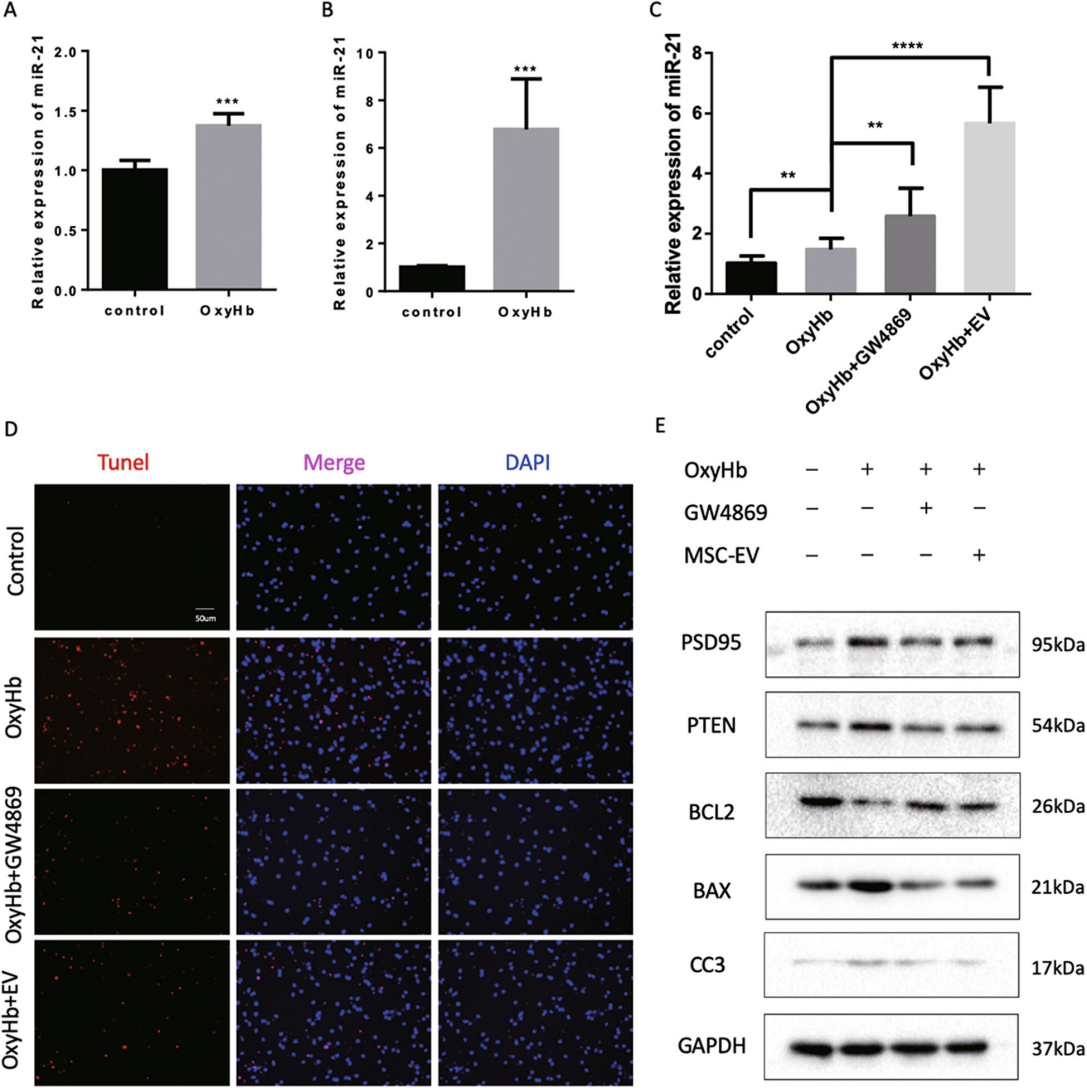
miR-21 was excreted out of neurons resulting in neuronal apoptosis. miR-21 was upregulated in cultured neurons after OxyHb insult (**a**), while EV-derived mir-21 increased more dramatically (**b**). Then, we applied EV secretion inhibitor GW4869 to explore the process. **c** GW4869 inhibited the secretion of mir-21 and decreased the amount of EV-derived mir-21. **d** GW4869 alleviated the apoptosis of OxyHb-treated neurons. **e** Western blot indicated that GW4869 could inhibit apoptosis after OxyHb treatment.

As previously mentioned, we speculated that miR-21-5p in neurons wore down through EV excretion. Thus, we examined the expression of miR-21-5p in cultured rat neurons and their conditioned medium (Fig. 7a and b). After 1-week in vitro culture, primary rat neurons formed mature synapses and were ready for oxyhemoglobin (OxyHb) treatment. We observed that miR-21-5p accumulated markedly in conditioned medium from OxyHb-treated (10 μmol/L) neurons than control group. We further employed GW4869 to inhibit EV secretion.

Administration of GW4869 increased the expression of miR-21-5p in neuronal cytoplasm (Fig. 7c) and inhibited neuronal apoptosis resulted from OxyHb treatment (Fig. 7d). As shown in Fig. 7e, western blotting also supported the anti-apoptosis effects of inhibiting EV secretion. Compared with MSC-EV, GW4869 treatment did not increase miR-21-5p that greatly but also inhibited neuronal apoptosis similarly. However, administration of GW4869 in vivo lack of targeting capacity and will disrupt many other EV-mediated beneficial biological activities, which limits its use in SAH-related cognitive dysfunction.

### MiR-21-5p targeted the PTEN/Akt axis in neurons to alleviate EBI after SAH

**Fig. 8:**
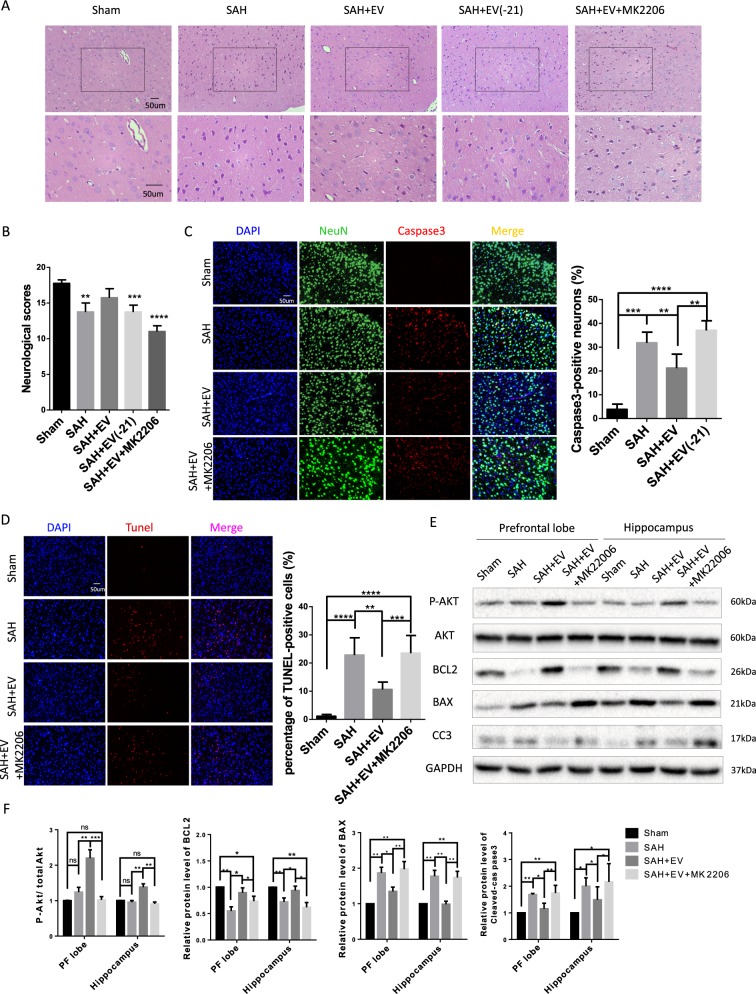
EV-derived miR-21 promoted neuronal survival after SAH through the PTEN/Akt signaling pathway. **a** Hematoxylin and eosin (HE) staining of the prefrontal cortex 48 h after SAH (*n* = 3). **b** Neurological behavior scores in each group (*n* = 6). **c** Double immunofluorescence staining of cleaved-caspase-3 and NeuN in the prefrontal cortex 48 h after SAH. **d** TUNEL staining. **e** Western blot. **f** The protein level of phospho-Akt was normalized to total Akt protein level and BCL2, BAX, and cleaved-caspase3 was normalized to that of GAPDH.

To further investigate the downstream mechanisms underlying the effects of EV-derived miR-21-5p, we used MK2206 to block AKT activation. MK2206 was administered at 100 μg per rat dissolved in DMSO through stereotaxic injection into lateral ventricle 2 days ahead of carotid perforation. In the presence of MK2206, MSC-EV could not effectively suppress brain injury and neuronal apoptosis induced by SAH (Fig. 8c–e). Furthermore, a modified Garcia scores test was performed to examine neurological function. As shown in Fig. 8a and b, both miR-21-5p inhibitors or MK2206 could block the neuroprotective effects of MSC-EV.

## Discussion

Many aneurysmal SAH patients suffer from cognitive function impairments even though they received proper treatment, such as the clipping or coiling of aneurysms, which causes problems in returning to work and burdens the family^3,35^. The severity of bleeding is associated with neuropsychological outcomes^36^. With anti-vasospasm therapy failing to improve outcomes in SAH patients, EBI that occurs in the first 72 h post SAH has drawn increasing attention in recent years and has emerged as an important risk factor for poor outcomes in SAH^7,37,38^. EBI is believed to be involved in pathophysiological changes post SAH, including global cerebral edema, ultraearly vasospasm, and reactive neuroinflammation^39^. Thus, EBI may be the root cause of cognitive dysfunction post SAH and needs to be addressed to improve the outcomes of SAH patients.

MSC transplantation has been used to treat rats suffering from SAH^17,18,40,41^. The therapeutic potential of MSCs in rodent models of SAH was documented for the first time in 2012. Khalili et al. demonstrated that the intravenous administration of MSC to rats that had been subjected to SAH improved the structural integrity of cerebral tissue to promote functional recovery^40,41^. In another study, bone marrow MSC (BMSC) administration alleviated SAH-induced EBI and ameliorated neurobehavioral impairments partly by inhibiting microglia activation^17^, which suggested that there is another mechanism that accounts for this functional protection. However, there are potential risks for using cell-based therapy, like developing tumors or immuno-rejection^42,43^.

Meanwhile, some studies showed that the therapeutic effects of MSC therapy were mediated by EVs secreted by MSCs rather than transdifferentiated MSCs^15,44^. In addition, the administration of MSC-derived EVs has comparable treatment effects as that of MSCs in rodent models of ischemic stroke and TBI^45^. Therefore, we hypothesized that MSC therapy might alleviate brain damage in SAH by secreting EVs loaded with biologically functional cargoes and that intravenous MSC-EV administration could recapitulate the response to therapy with the secreting cells.

A few studies have reported miRNA changes after SAH^19,46^, but its regulatory role remain largely unclear. We demonstrated that protective miR-21-5p increased reactively in neurons upon SAH induction and wore down through EV secretion, which led to neuronal apoptosis. Moreover, inhibiting EV secretion can restrain more miR-21-5p in neurons. However, GW4869 administration in vivo will disrupt many other benneficial cell-to-cell communications and may produce side effects. Fortunately, according to many researches, MSC-EV could target injured neurons to deliver abundant miRNA. Thus, we employed MSC-EV enriched with miR-21-5p to replenish miR-21-5p in SAH-insulted neurons. Both in vivo and in vitro, MSC-EV successfully increased miR-21-5p in brain or neurons and protected more neurons form apoptosis, and then improve the cognitive function of SAH rats.

Pten/Akt signaling has been implicated in SAH pathogenesis. S473 Phosphorylation of Akt can protect neuron from death^47^, while the upstream regulator PTEN inhibited neuronal survival^28,48^. In this study, we demonstrated that PTEN was upregulated in OxyHb-treated neurons and prefrontal cortex of SAH-insulted rats and EV-derived miR-21-5p from mesenchymal stem cells could significantly inhibited PTEN expression to protect neuronal apoptosis and improve cognitive function.

We employed EV sequencing to identify cargoes the EVs carried and found that miR-21-5p, along with the other top 6 miRNAs, constitutes nearly 60% of all miRNA contents. miR-21-5p alone accounts for as much as 22.5% of miRNA contents. Interestingly, an increase in miR-21-5p was also found in CSF from SAH patients and was correlated with DCI occurrence^19^. As is well known, the PTEN/Akt signaling pathway is regulated by miR-21-5p and promotes cell survival in various diseases, including SAH^49–51^. Phospho473-Akt levels decreased significantly during EBI after SAH^52,53^. However, Hidenori et al. reported that Akt phosphorylation was accelerated in cortical and hippocampal neurons after SAH, especially in the first 6 h. However, at 24 h post SAH, Akt phosphorylation decreased significantly^47^. Furthermore, both previous studies and our preliminary study^23^ showed that the overall protein level of PTEN increased and the pharmaceutical inhibition of PTEN could protect against EBI after SAH^28,54^. Thus, we proposed that Akt signaling was activated in response to SAH onset, but that its activity could not be sustained to protect neurons and that miR-21-5p as an upstream regulator may be involved in the process. Further studies could reveal the detailed mechanisms involved in SAH pathogenesis to develop new therapies based on the miR-21-5p/PTEN/Akt signaling pathway. Using qRT-PCR, we observed that the expression level of miR-21-5p in the prefrontal cortex and hippocampus first increased after SAH yet decreased afterward, implying that miR-21-5p in CSF may originate from neurons and interrupting the discharge of miR-21-5p or replenishing miR-21-5p in neurons may help protect neurons against SAH-induced EBI via PTEN/Akt signaling.

In this study, we identified the protective role of MSC-derived EVs in a rat model of SAH. MSC-EV improved neurological scores significantly and alleviated brain edema resulting from SAH. Using EV tracing, we verified that MSC-EV was taken up by damaged neurons to exert its effects. In addition, a TUNEL assay and cleaved-caspase-3/NeuN double staining showed a decrease in apoptosis in the SAH + EV group compared with the SAH + PBS group, which was also confirmed by Western blot. MSC-EV increased Bcl-2 expression and inhibited Bax and Caspase-3 expressions in both prefrontal lobe and hippocampus, suggesting that it suppressed cellular apoptosis through inhibiting the mitochondrial apoptosis pathway. To investigate the mechanism involved in the neuroprotective effects of MSC-EV in SAH, we employed miRNA EV sequencing to identify the specific molecular cargo. According to the online data from exRNA Atlas, we assumed that miR-21-5p is the key target that mediates the therapeutic effects.

Thus, miR-21-5p inhibitors and MK2206 were used to explore the involved mechanism, either of which was sufficient to impair the effects of MSC-EV. Furthermore, we showed that SAH rats treated with MSC-EV exhibited better cognitive function in the Morris Water Maze test than those treated with PBS. In brief, we demonstrated that EV-derived miR-21-5p alleviated brain injury after SAH through inhibiting neuronal apoptosis and improved the neurological behaviors via the PTEN/Akt pathway, which provides support for a potential new restorative therapy for SAH patients at risk for cognitive defects.

The current study possesses some limitations and could be improved in several aspects. First, EVs can be decorated with targeting peptides to promote delivery efficiency. For example, rabies virus glycoprotein (RVG) peptide can be fused to the EV-derived membrane protein Lamp2b to enhance targeting capability in the brain^55^. In subsequent studies, RVG EVs were proven to be effective for miRNA or siRNA delivery to the brain for disease treatment^56,57^. In some studies, naive EVs were loaded with additional therapeutic agents to achieve better therapeutic effects. For example, EVs loaded with curcumin or cucurbitacin I suppressed inflammatory responses in models of lipopolysaccharide (LPS)-induced brain inflammation, experimental autoimmune encephalitis, and GL26 brain tumors, respectively^58^. Furthermore, the therapeutic potential of EVs carrying excess miRNA has been investigated. In a rat model of middle cerebral artery occlusion, miR-133b-rich EVs promoted neurite remodeling and functional recovery^15,16^. In a subsequent study, they also demonstrated that engineered MSC EVs containing elevated miR-17–92 clusters had therapeutic benefits for stroke^44^.

However, most studies mentioned above did not employ antibodies of classic EV markers, such as CD63 or CD81, to isolate EVs, which leads to the isolation of a mixture of microvesicles shed from the plasma membrane, endosome-derived real EVs and nonvesicular extracellular nanoparticles^59^. The molecular constituents of these extracellular components differ greatly from each other. Further exploration may help to identify the specific contents that produce therapeutic benefits in animal models of various diseases.

In conclusion, we found that MSC-EV alleviated brain injury after SAH through inhibiting neuronal apoptosis and improved the neurological behavior, which expands the previous understanding of MSC-based therapy for SAH and provides evidence for a potential new restorative therapy that could be used for SAH patients at risk of cognitive defects.

## Supplementary information

Supplementary Figure Legends

Supplementary Figure 1

Supplementary Figure 2

Supplementary Figure 3

Supplementary Figure 4

Supplementary Figure 5

Supplementary Figure 6

Supplementary Figure 7

Supplementary Figure 8

## Data Availability

Because some of this research is still in progress, the raw data cannot be shared at present.
