# Supplementary material for: Extracellular vesicle-mediated transfer of miR-21-5p from mesenchymal stromal cells to neurons alleviates early brain injury to improve cognitive function via the PTEN/Akt pathway after subarachnoid hemorrhage
Source: Cell Death Dis. 2020 May 13;11(5):363. doi: 10.1038/s41419-020-2530-0 (PMC7220929; doi:10.1038/s41419-020-2530-0)
Supplement: Supplementary file 1 — Supplementary Figure Legends [file 41419_2020_2530_MOESM1_ESM.docx]

Supplementary Figure Legends

Fig. S1. The identification and quantification of MSC-derived Extracellular vesicles.

A. qNano scanning of isolated MSC-derived exosomes showing that particle sizes distribute mainly at 50-200nm.

B. Protein level of TSG-101 and CD9 in extracellular vesicle compared with cell lysate. And the endoplasmic reticulum marker Calnexin was also used as control.

C. Representative image of cultured MSC in light field.

Fig. S2. Representative images of control group for PKH67-EV tracing assay.

Fig. S3. Representative images of PKH67-labelled extracellular vesicle tracing stained with NeuN or GFAP antibody in prefrontal lobe(A) and hippocampus(B).

Fig. S4. Representative images of PKH67-labelled extracellular vesicle tracing in kidney, liver and spleen.

Fig. S5. Representative images of Tunel staining in dentate gyrus treated with or without MSC-EV.

Fig. S6. Dot plot showing the differential expression levels of let-7i-5p, let-7f-5p and let-7b-5p in human CSF.

Fig. S7. Relative expression of top miRNAs from extracellular vesicle sequencing at 48h post SAH of both prefrontal cortex and hippocampus, including let-7i, let-7c, miR-26a, let-7f, let-7b and miR-99a.

Fig. S8. miR-21 targeted PTEN rather than Rela or PDCD4 *in vitro*.
