# Supplementary figures and images for: Extracellular vesicle-mediated transfer of miR-21-5p from mesenchymal stromal cells to neurons alleviates early brain injury to improve cognitive function via the PTEN/Akt pathway after subarachnoid hemorrhage
Source: Cell Death Dis. 2020 May 13;11(5):363. doi: 10.1038/s41419-020-2530-0 (PMC7220929; doi:10.1038/s41419-020-2530-0)

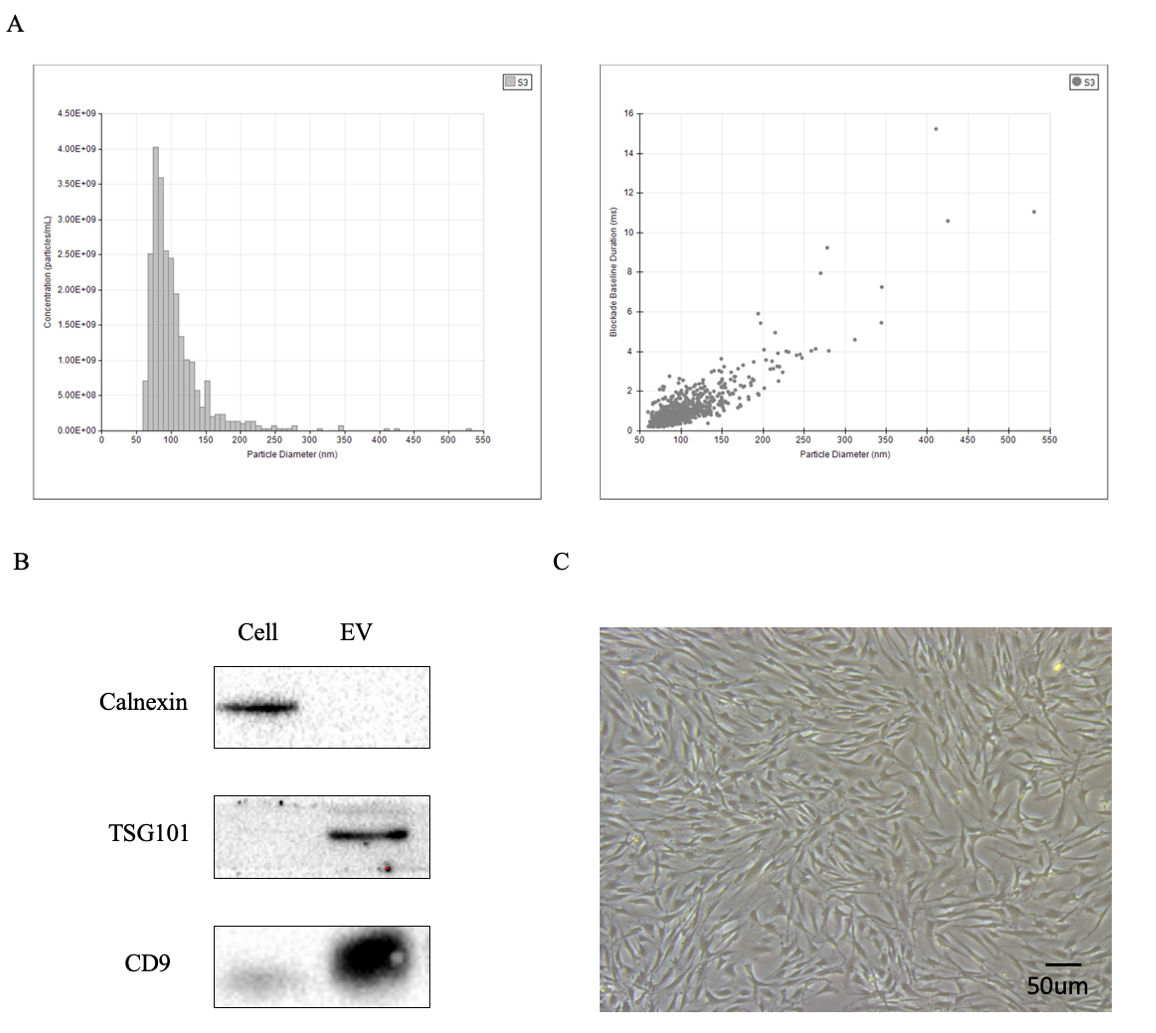

Supplement: Supplementary file 2 — Supplementary Figure 1 [file 41419_2020_2530_MOESM2_ESM.png]

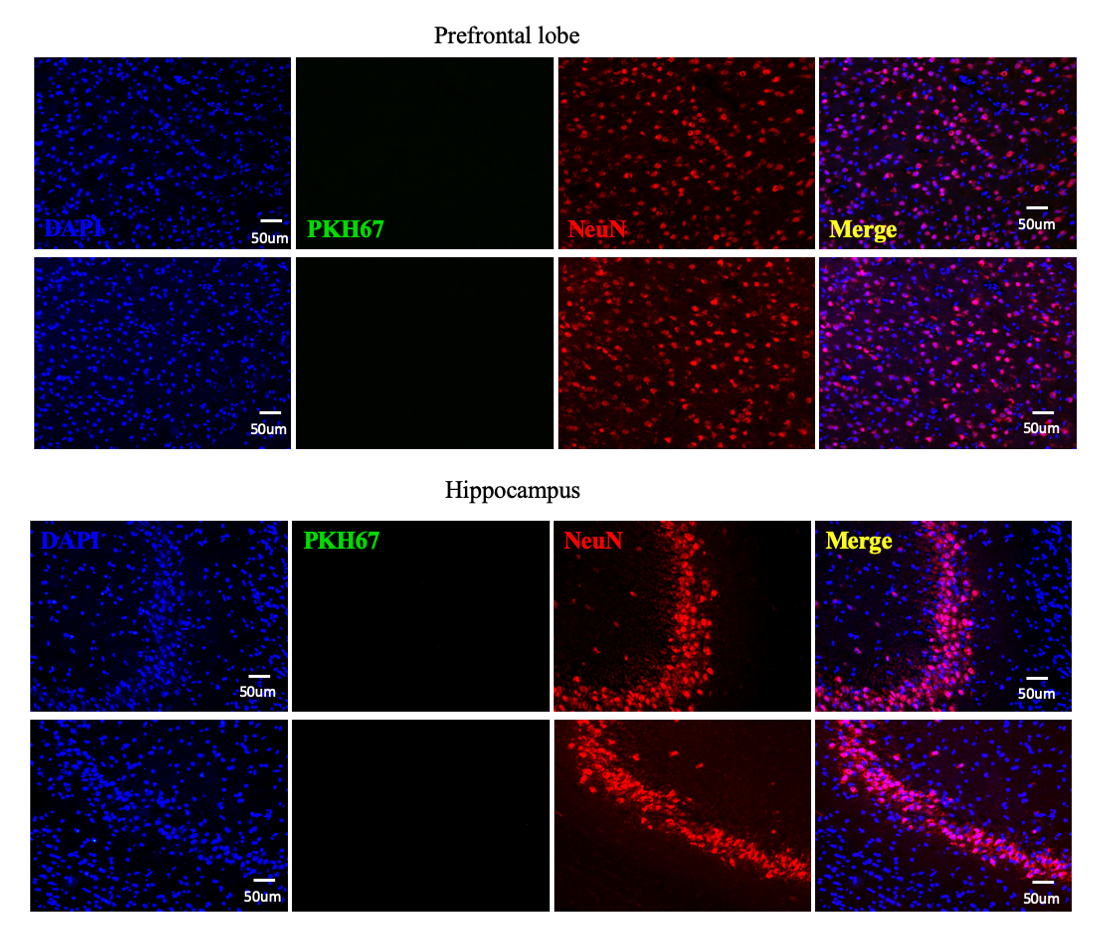

Supplement: Supplementary file 3 — Supplementary Figure 2 [file 41419_2020_2530_MOESM3_ESM.png]

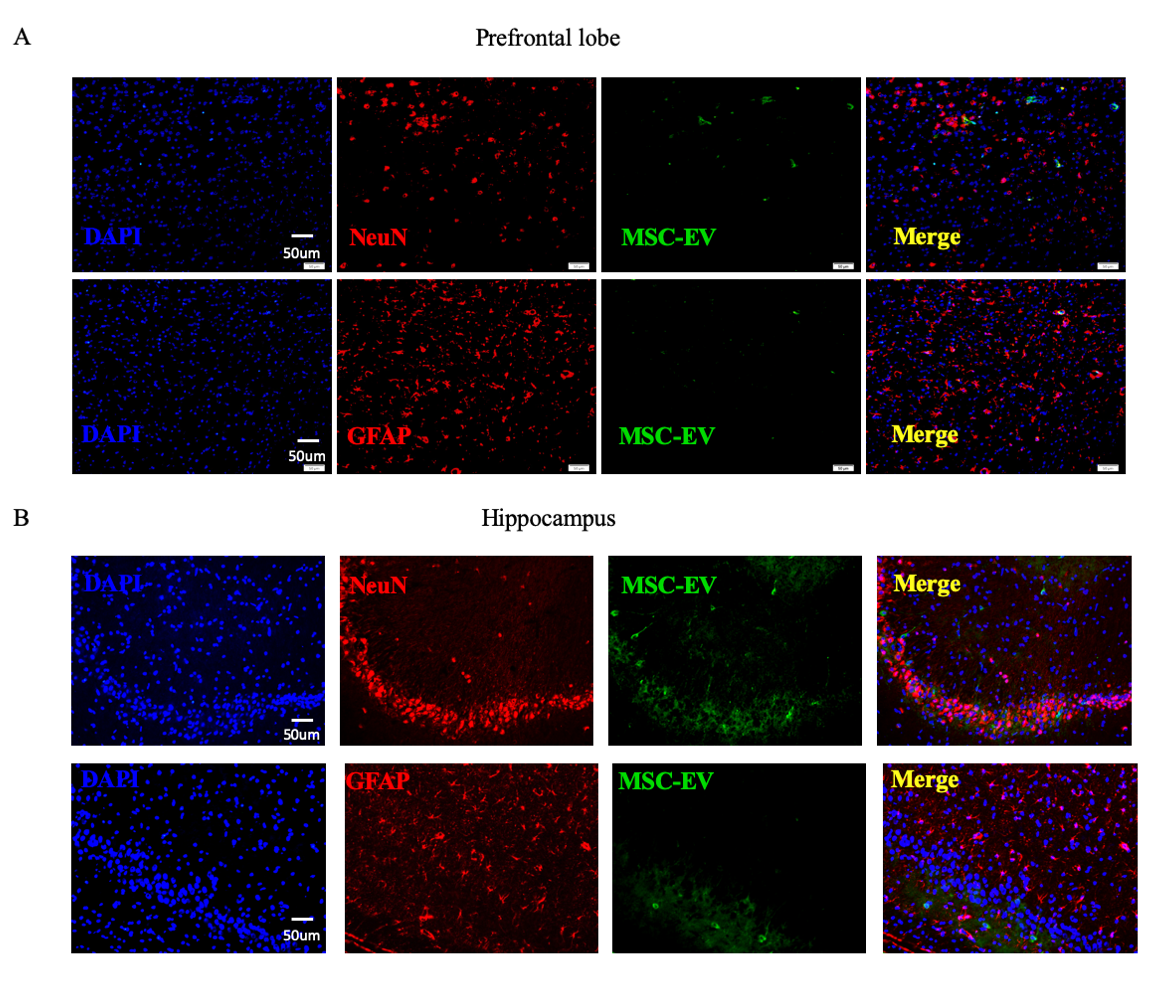

Supplement: Supplementary file 4 — Supplementary Figure 3 [file 41419_2020_2530_MOESM4_ESM.png]

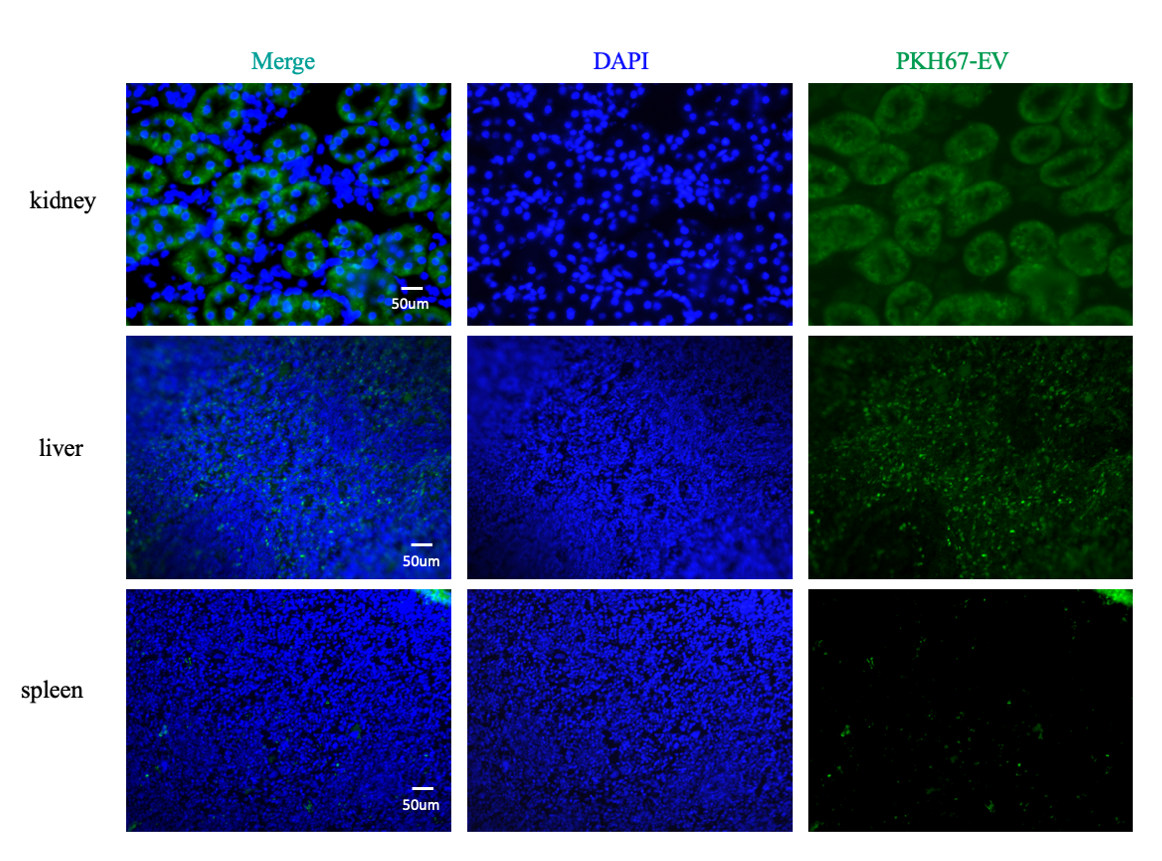

Supplement: Supplementary file 5 — Supplementary Figure 4 [file 41419_2020_2530_MOESM5_ESM.png]

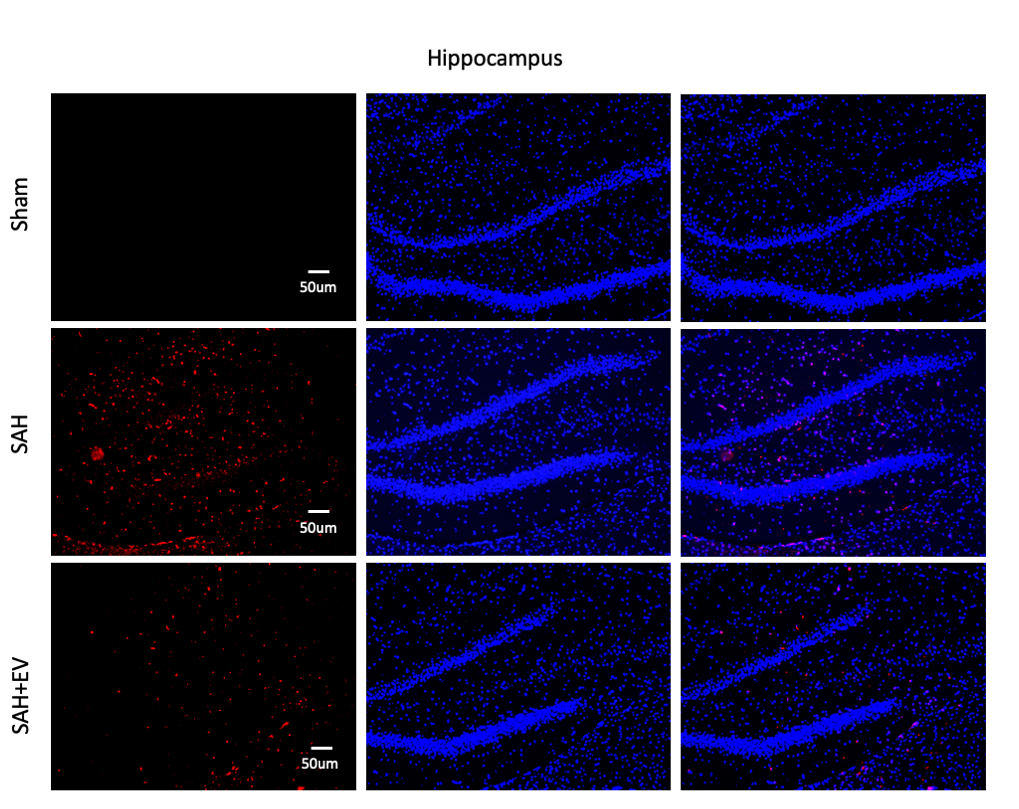

Supplement: Supplementary file 6 — Supplementary Figure 5 [file 41419_2020_2530_MOESM6_ESM.png]

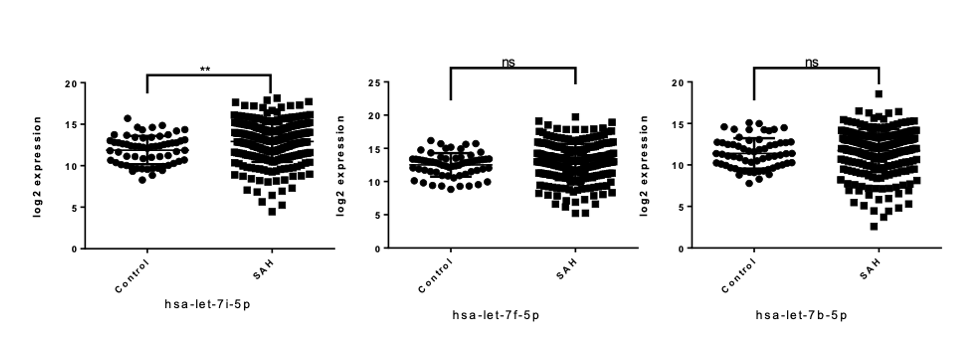

Supplement: Supplementary file 7 — Supplementary Figure 6 [file 41419_2020_2530_MOESM7_ESM.png]

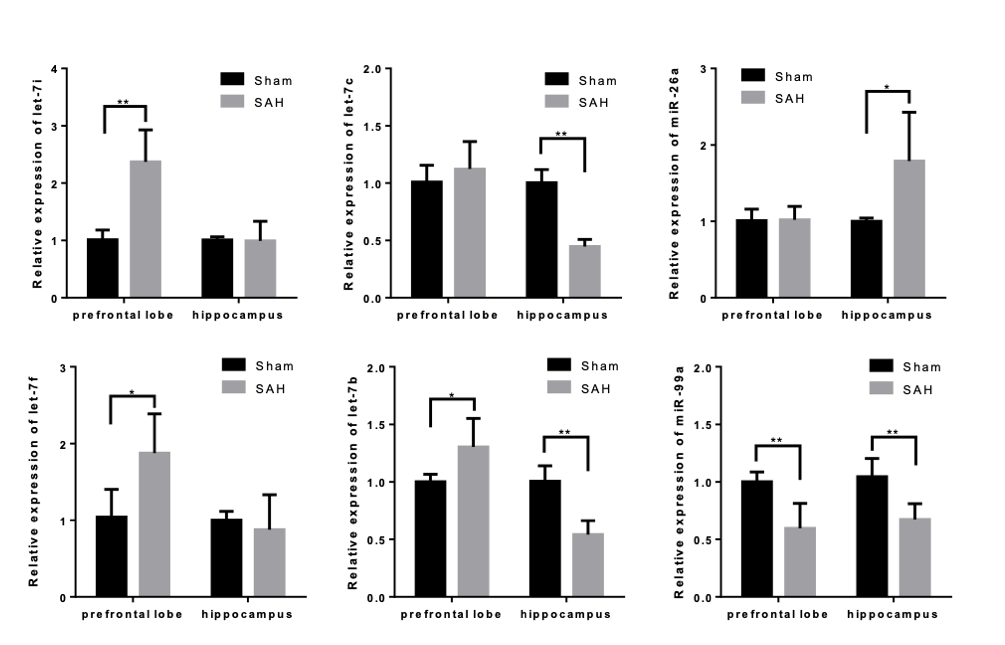

Supplement: Supplementary file 8 — Supplementary Figure 7 [file 41419_2020_2530_MOESM8_ESM.png]

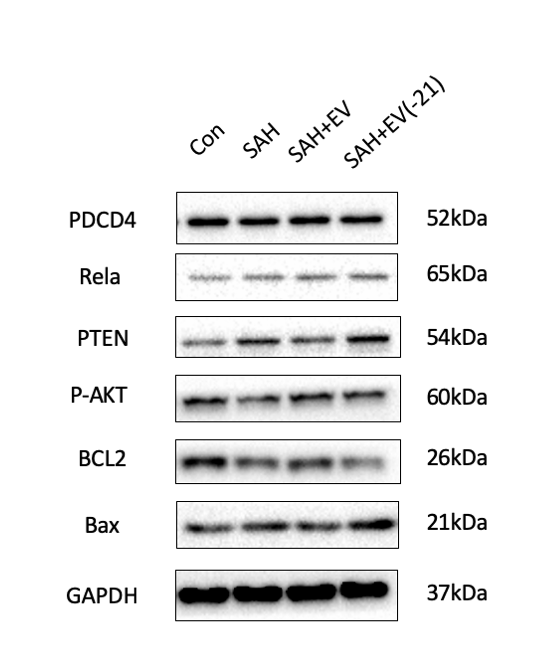

Supplement: Supplementary file 9 — Supplementary Figure 8 [file 41419_2020_2530_MOESM9_ESM.png]
